# A Multigranularity Text Driven Named Entity Recognition CGAN Model for Traditional Chinese Medicine Literatures

**DOI:** 10.1155/2022/1495841

**Published:** 2022-09-24

**Authors:** Yuekun Ma, Yun Liu, Dezheng Zhang, Jiye Zhang, He Liu, Yonghong Xie

**Affiliations:** ^1^School of Computer and Communication Engineering, University of Science and Technology Beijing, Beijing 100083, China; ^2^School of Artificial Intelligence, North China University of Science and Technology, Tangshan 063210, China; ^3^Beijing Key Laboratory of Knowledge Engineering for Materials Science, Beijing 100083, China; ^4^Beijing Haidian Hospital, Beijing 100080, China

## Abstract

Recognition of Traditional Chinese Medicine (TCM) entities from different types of literature is challenging research, which is the foundation for extracting a large amount of TCM knowledge existing in unstructured texts into structured formats. The lack of large-scale annotated data makes unsatisfactory application of conventional deep learning models in TCM text knowledge extraction. Some other unsupervised methods rely on other auxiliary data, such as domain dictionaries. We propose a multigranularity text-driven NER model based on Conditional Generation Adversarial Network (MT-CGAN) to implement TCM NER with small-scale annotated corpus. In the model, a multigranularity text features encoder (MTFE) is designed to extract rich semantic and grammatical information from multiple dimensions of TCM texts. By differentiating the conditional constraints of the generator and discriminator of MT-CGAN, the synchronization between the generated tag labs and the named entities is guaranteed. Furthermore, seeds of different TCM text types are introduced into our model to improve the precision of NER. We compare our method with other baseline methods to illustrate the effectiveness of our method on 4 kinds of gold-standard datasets. The experiment results show that the standard precision, recall, and F1 score of our method are higher than the state-of-the-art methods by 0.24∼8.97%, 0.89∼12.74%, and 0.01∼10.84%. MT-CGAN is able to extract entities from different types of TCM literature effectively. Our experimental results indicate that the proposed approach has a clear advantage in processing TCM texts with more entity types, higher sparsity, less regular features, and a small-scale corpus.

## 1. Introduction

Named Entity Recognition (NER) in Traditional Chinese Medicine (TCM) texts is an important task of TCM knowledge extraction, which refers to extracting the instances of TCM domain concepts from large-scale unstructured TCM texts and identifying their concept types. The grammatical form of classical Chinese and semantic peculiarities of TCM texts leads to the complexity of NER in the TCM field being higher than in other fields.

At present, there are deficiencies in various mainstream NER methods for general domain texts and TCM texts. Since the domain characteristics of TCM texts are not considered, the NER methods of generic domain texts have weak generalization ability and unsatisfactory recognition results in TCM texts. Compared with other methods, the NER models based on deep learning in the field of TCM texts have achieved better results, but they tend to need a large-scale training corpus. The highly specialized nature of TCM texts leads to the high cost of training corpus annotations. The lack of large-scale annotated data makes unsatisfactory application of conventional deep learning models in TCM text knowledge extraction. How to use the limited annotated corpus to realize NER for massive TCM texts has become an urgent problem to be solved.

Researchers have actively explored in NER domain with a lack of annotation data. Jia et al. [1] considered NER in TCM as a text span detection task, classifying all text spans and obtaining the relevant knowledge entities from the sentences based on the classification results. The approach does not require a large-scale annotated corpus but is based on a high-quality domain dictionary. Han Yuanbo Tao [2] combined Generative Adversarial Networks (GAN) with the BiLSTM-Attention-CFR model to solve the problems of lack of annotation data for domain NER tasks and inconsistent annotation of entities in the same document. However, this model requires a large-scale crowdsourced annotated dataset, although the cost of the crowdsourced annotation dataset is lower than that of a professional annotation dataset.

To solve the above problems, this paper introduces the generative adversarial idea to the NER tasks and proposes a Multigranularity Text Features Fused NER model based on CGAN (MT-CGAN). The model takes advantage of the fact that the Conditional Generation Adversarial Network (CGAN) model can generate data with the same distribution characteristics as the real data [3], and self-attention is better at capturing the internal correlation of data or features. Using this model, we extract features at different granularities of TCM texts and improve the domain NER performance under the condition of a small amount of annotated corpus. We summarize the contribution as follows:

- A differential feature-constrained NER model in TCM texts is proposed based on the generative adversarial idea. The model solves the problems of lack of annotation data in TCM texts and the inapplicability of NER models in other domains in the NER tasks of TCM texts.
- A Multigranularity Text Features Encoder (MTFE) is designed to extract grammatical and semantic features embedded in different dimensions and feature spaces of text from words, sentences, paragraphs, and chapters, respectively, to enhance the multidimensional feature learning capability of the model.
- An improved U-NET is added to the generator of the MT-CGAN to hierarchically extract the features of the seed label. The extracted features are also passed to the decoding layer via jump links to achieve feature enhancement of the generation process. This further reduces the randomness of the generated content and speeds up the convergence of the model.

The paper is organized as follows.  Section 2 introduces the work related to the NER tasks in TCM texts and multigranularity features extraction.  Section 3 describes the details of the MT-CGAN.  Section 4 describes the experiments for the performance evaluation of the MT-CGAN.  Section 5 concludes the paper.

## 2. Related Work

### 2.1. NER of TCM Texts Based on Deep Learning

Now, deep learning has become a mainstream technique for solving NER tasks because of its ability to discover hidden features. Deng et al. [4] combined BiLSTM and CRF and applied it to the Chinese medicine text NER, and the model improved the accuracy of NER because of the powerful ability of the LSTM model to extract global contextual semantic information. Li et al. [5] proposed a model called BiLSTM-Att-CRF by integrating attention into BiLSTM networks and proved that this model can avoid the problem of information loss caused by distance. An et al. [6] proposed a Multihead Self-attention-based BiLSTM-CRF model (MUSA-BiLSTM-CRF) for Chinese clinical named entity recognition. The proposal of the Transformer model [7] opened the era of dominance of pretrained models in NLP [8, 9]. As a typical representative of pretrained models, BERT [10] has shown good performance in many NLP tasks [11, 12]. Qu et al. [13] applied the combination of the BERT model and BiLSTM-CRF to the NER task of TCM texts, and the performance was improved compared with the BiLSTM-CRF model. Qu et al. [13] constructed a named entity recognition model based on Bert-BiLSTM-CRF to solve the problem of fuzzy entity recognition and less labeled data in the field of traditional Chinese medicine. Zhang et al. [14] proposed a semisupervised embedded Semi-BERT-BiLSTM-CRF model. Xiong et al. [15] proposed a method based on a relational graph convolutional network (RGCN), which utilized multisource knowledge in a unified manner for Chinese Clinical NER. All of these NER models employed large-scale annotated samples or pretrained models to maximize performance.

Compared with other deep learning models with end-to-end training, GAN can learn the distribution of real samples and has better robustness, which can alleviate the problem of insufficient labeled samples in deep learning.

### 2.2. Conditional Generation Adversarial Network

Generative Adversarial Networks (GAN) is a generation framework. Because it can use the adversarial sample [16] to generate data with the same distribution as the real target, it is applied to a variety of generation tasks. For example, Cao [17] used it to restore images and graphs. The original GAN has some limitations, which are mainly reflected in the uncontrollable process of generator generating samples. For this reason, Mirza and Simon [18] proposed CGAN. It aims to change the random generation of the target object and control the data generation process by incorporating specific conditions into the generator and discriminator inputs. It solves the problem that GAN cannot generate specific attributes in the NLP. Feng [19] proposed a Conditional Wasserstein Generative Adversarial Network model (CWGAN) for NER tasks by combining CGAN and a modified Wasserstein Generative Adversarial Network (WGAN-GP). They verified the effectiveness of the CWGAN on the generic domain NER tasks. However, this model does not perform well in the NER task of TCM texts.

At the same time, by analyzing the characteristics of current NER models based on the generative adversarial idea, we find that these models are all based on feature extraction from a single granularity of text. As the carrier of knowledge, text with different granularity contains different information. Text features can be mined more comprehensively through multigranularity text feature extraction.

### 2.3. Multigranularity Features Extraction

It was found that a multigranularity cognitive mechanism was used to improve the NER task. Zhang and Yang [20] designed a Lattice-LSTM model to encode characters and words, respectively, which achieved the utilization of information on both words and order of words and avoided the problem of word separation errors. Gao et al. [21] proposed contextual multifeature embedding (CMFE) and used it to construct a multifeature semantic fusion model (MFSFM) to realize citation entity recognition. Sun [22] referred to a method of recognizing Chinese clinically named entities based on multistrategy fusion. Li et al. [23] proposed a Flat-Lattice Transformer model, which transformed the Lattice structure into Flat-Lattice to link the semantic information of characters and words together for NER.

Multigranularity feature extraction can obtain richer semantic information than single-granularity feature extraction. In the NER tasks, whether the semantic information can be fully extracted is the key to the performance of the model. Therefore, we propose the MT-CGAN to solve the problem of NER in TCM texts.

## 3. Model

In this paper, we view the NER tasks in TCM as sequence annotation tasks and use the generative adversarial idea to construct the MT-CGAN model for implementing NER in TCM texts in a small-scale corpus. The description of the tasks is as follows: given an annotated corpus text, *X*={*x*_0_, *x*_1_,…, *x*_*i*_,…, *x*_*n*_} is the text sequences, *L*={*l*_0_, *l*_1_,…, *l*_*i*_,…, *l*_*n*_} is the sequence of named entities labels for the corresponding text sequence *X*, where the *x*_*i*_ represents a character, and *x*_*i*_ represents the entity category label corresponding to *x*_*i*_. The role of the generator of the model is to generate the sequence of annotations *L*′={*l*_0_′, *l*_1_′,…, *l*_*i*_′,…, *l*_*n*_′} corresponding to. The discriminator guides the model training by scoring *L*′ so that the generator can generate a sequence of entity labels that approximates *L*. The following is a detailed description of the model.

### 3.1. Model Structure

**Figure 1:**
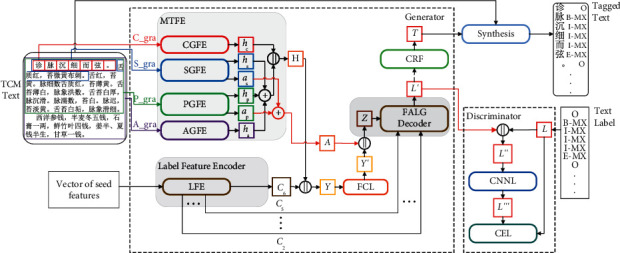
Model framework. || is a splicing operation, + is a contraposition addition operation. C_gra, S_gra, P_gra, and A_gra denote characters, sentences, paragraphs, and chapters of text, respectively. FCL represents a fully connected layer. CNNL represents the convolutional neural network Layer, and CEL denotes the cross-entropy loss function.

The MT-CGAN model proposed in this paper consists of the generator and the discriminator, and the structure is shown in Figure 1. The generator of the MT-CGAN consists of the MTFE and a modified U-NET (conditionally enhanced U-NET, C-U-NET). The discriminator of the MT-CGAN consists of multiple Convolutional Neural Networks (CNN) [24], normalization operations of each layer, and activation functions.

In this paper, the text attention data generated by the MTFE is used to replace the white noise input of the CGAN generator as the main input of the model generator in the text feature extraction process. Moreover, to improve the quality of label sequence generation, we fuse the multigranularity text features extracted by the MTFE with the TCM text entity distribution features extracted by the encoder of the C-U-NET and as the conditional input *Y* to the model's generator.

In the discriminator part of the MT-CGAN, we use a small-scale of real sample labels *L* as the conditional input of the discriminator, which improves the ability of the model to discriminate authenticity and accelerates the convergence speed of the model training.

The generator of the MT-CGAN generates a sequence of entity labels in vector form, and a Conditional Random Field (CRF) [25] is added to which to convert the vector into an intuitive symbolic representation form. When the MT-CGAN training reaches a steady state, the model loads the trained generator model to generate a sequence *L*′ of text labels. Then *L*′ is used as the input for the CRF to obtain the mapping of text labels *T*. Finally, the generated text labels *T* are synchronized with the corresponding text to form the final text annotation sequence.

### 3.2. Multigranularity Text Features Encoder

The MTFE of the MT-CGAN consists of four feature encoders with different granularity of characters, sentences, paragraphs, and chapters to extract text features from different dimensions.

The Character-Granularity Feature Encoder (CGFE) is implemented based on the GRU [26] network and is used to extract the position information *h*_*c*_ among characters in text. The Sentence-Granularity Feature Encoder (SGFE) is realized based on Transformer's encoder for extracting text features *h*_*s*_ embedded in the textual context within a sentence. The Paragraph-Granularity Feature Encoder (PGFE), also based on Transformer's encoder, is introduced into this model to extract the deeper latent semantic and syntactic features *h*_*p*_ contained among different sentences within a text segment. A local central feature within a chapter is important to the NER in TCM texts, so the Article-Granularity Feature Encoder (AGFE) based on CNN is designed to achieve the extraction of central features *h*_*a*_ from the global scope of the chapter text.

The SGFE and PGFE adopt the attention mechanism that can generate attention data for the corresponding granularity of text while extracting deep semantic features of the text context. Because these data reflect the contextual relevance among tokens, we fused the attention data generated by the SGFE and PGFE to form the main input to the generator of the MT-CGAN.

#### 3.2.1. Character-Granularity Feature Encoder

To ensure that the generated sequence of text labels is consistent with the original text sequence, this paper designs the CGFE based on GRU to extract the relative position feature *h*_*c*_ of characters in TCM texts and uses *h*_*c*_ to constrain the order of the entities label generating.

**Figure 2:**
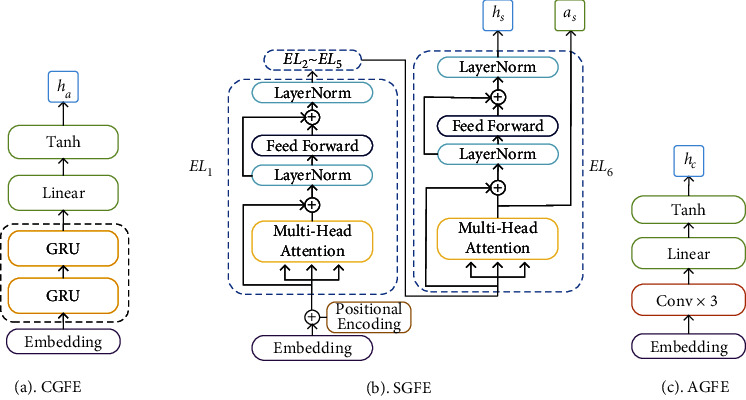
Network structure of each component of the MTFE. (a) CGFE (b) SGFE (c) AGFE.

As shown in Figure 2(a) CGFE consists of an embedding layer, GRU, a fully connected layer, and an activation function. The GRU that can retain information in a sequence for long periods is used to encode character position information in text. It includes a reset gate and an update gate. The extent to which the preceding information is retained is determined by both. The smaller the value of the reset gate is, the more previous information is discarded, while the larger the value of the update gate, the more is retained.

The equation of reset gate is as follows:

(1) $$\begin{array}{c} r_{i}=\sigma \left(W^{\left(r\right)}e_{i}+U^{\left(r\right)}p_{i-1}\right), \end{array}$$

where *r*_*i*_ is the reset gate, *e*_*i*_ is the vector of the current input token, *W*^(*r*)^ and *U*^(*r*)^ are the reset gate weight matrices, and *p*_*i*−1_ is the relative position information of the previous token.

The equation of the update gate is as follows:

(2) $$\begin{array}{c} z_{i}=\sigma \left(W^{\left(z\right)}e_{i}+U^{\left(z\right)}p_{i-1}\right), \end{array}$$

where *z*_*i*_ is the gating controlling the update gate, *W*^(*z*)^ and *U*^(*z*)^ are the weight matrices of the update gate.

The linear transformation parameters of the update gate and the reset gate are changed in the MT-CGAN model to obtain the candidate hidden state *p*_*i*_′ and the output *p*_*i*_, respectively. *p*_*i*_′ and *p*_*i*_ are calculated by (3) and (4), respectively.

(3) $$\begin{array}{c} p_{t}^{\prime }=\text{Tanh}\left(We_{i}+r_{i}⊙Up_{i-1}\right), \end{array}$$

where ⊙ is the Hadamard product and the data is mapped between (−1, 1) by the Tanh activation function.

(4) $$\begin{array}{c} p_{i}=\left(1-z_{i}\right)⊙p_{i-1}+z_{i}⊙p_{i}^{\prime }, \end{array}$$

where (1 − *z*_*i*_)⊙*p*_*i*−1_ represents the forgetting of the relative position information of the previous character and *z*_*i*_⊙*p*_*i*_′ represents the remembering of the current position information.

The text character sequence passes through two GRU layers to obtain the character relative position feature sequence *P*:

(5) $$\begin{array}{c} P=p_{1},p_{2},\ldots ,p_{i},\ldots ,p_{n}. \end{array}$$

After the full concatenation and Tanh activation operations, the final character position feature *h*_*c*_ of the text sequence is obtained.

(6) $$\begin{array}{c} h_{c}=\left\{h_{c}^{0},h_{c}^{1},\ldots ,h_{c}^{i},\ldots ,h_{c}^{n}\right\}, \end{array}$$

#### 3.2.2. Sentence-Granularity and Paragraph-Granularity Feature Encoder

The entities in the field of TCM texts have their own characteristics. According to Yurui [27], concepts in the field of TCM texts are characterized by heterogeneity, and they have obvious multimeaning and fluidity, and the same concept could have different functions. The boundaries between the categories of entities, attributes, and relations are ambiguous. For example, the word 经脉 appears 29 times throughout the text of *Qian Jin Fang*, and its connotation and scope vary in different contexts, indicating the meanings of Channel of Qi-Blood, Channel Number, and Menses, respectively. Therefore, to achieve accurate NER on TCM texts, the context of the text must be fully used.

The contextual semantic spaces constructed within a sentence and among different sentences are different, and the different semantic spaces have different representations for TCM knowledge and have different roles for NER in the TCM domain. The token level and sentence level semantics are equally important. Therefore, it is necessary to obtain deep-level potential semantic features of text from different scope contexts by using text of both sentence and paragraph granularities as objects.

Transformer can find latent recognition patterns from data and has been proven to have the ability to extract deep semantic features embedded in a text [28]. To benefit from its self-attention mechanism, the Transformer can give the same attention to all elements of the input text sequence and can understand the relationship among different elements of input sequences, although the elements may be far away from each other [29]. Transformer models can model the relationships among words in long sentences or paragraphs, so the sentence-granularity and paragraph-granularity text feature encoders proposed in this paper are both implemented based on the encoder of Transformer.

In the following, we use the SGFE as an example to illustrate how attention data and contextual features of text are extracted based on Transformer. As shown in Figure 2(b), the SGFE is composed of a stack of six identical encoding layers *EL*_*i*_, where *i*=1,…, 6 and represents the index of the encoding layers. We can obtain *a*_*i*_ and *h*_*i*_ through each encoding layer. *a*_*i*_ and *h*_*i*_ represent the attention data and contextual feature data, respectively. The outputs of the SGFE are *a*_6_ and *h*_6_ obtained from *EL*_6_. Each encoding layer has two sublayers. The first is a multihead self-attention mechanism, and the second is a fully connected feedforward network. The input of *EL*_1_ is a vector matrix *E*_*s*_={*e*_*s*_^0^,…, *e*_*s*_^*i*^,…, *e*_*s*_^*n*^}, *e*_*s*_^*i*^ denotes the vector corresponding to the *i*th word in the input text sequence.

The following describes how the attention data and contextual feature data of a text sequence are calculated.

*(1) Attention Data.* Multihead self-attention consists of 8 attention layers running in parallel, each attention layer calculates different attention data fragments of the input text from different semantic spaces, and then the eight fragments are concatenated to form the attention data. The equation for calculating the attention data fragment is as follows:

(7) $$\begin{array}{c} Z_{h}=\text{softmax}\left(\frac{Q_{h}\times K_{h}}{\sqrt{d_{k}}}\right)\times V_{h}, \end{array}$$

where *Q*_*h*_ represents the query matrix, *K*_*h*_ represents the key matrix, *V*_*h*_ represents the value matrix obtained by transforming *E*_*s*_ through the linear matrices *W*_*h*_^*Q*^ ∈ ℝ^*d*_*k*_×*d*_*mo*  *de*  *l*_^, *W*_*h*_^*K*^ ∈ ℝ^*d*_*k*_×*d*_*mo*  *de*  *l*_^ and *W*_*h*_^*V*^ ∈ ℝ^*d*_*v*_×*d*_*mo*  *de*  *l*_^ respectively, and *h* represents the index of the attention header, *d*_*k*_=*d*_*v*_.

The output data of the multihead self-attention are concatenated by each attention data fragment according to the following:

(8) $$\begin{array}{c} a_{i}=\text{concat}\left(Z_{1},Z_{2},\ldots ,Z_{8}\right)\times W_{O}, \end{array}$$

where *W*_*O*_ ∈ ℝ^*hd*_*v*_×*d*_*mo*  *de*  *l*_^ is the matrix weight.

*(2) Contextual Feature Data.* The calculation of the output *h*_*i*_ for *EL*_*i*_ is as follows:

(9) $$\begin{array}{c} h_{i}=\text{LayerNorm}\left(\left(\text{LayerNome}\left(a_{i}+f\right)\right)+\text{Linear}\left(\text{LayerNome}\left(a_{i}+f\right)\right)\right), \end{array}$$

where *Linear* is the fully connected operation, *LayerNorm* represents the layer normalization operation, when *i*=1, *f* represents the vector matrix *E*_*s*_, and when *i*=2,…, 6, it represents the output of the previous encoding layer *h*_*i*−1_.

#### 3.2.3. Article-Granularity Feature Encoder

Usually, different TCM articles focus on different themes. The core sentence of a chapter is considered the carrier of the overall idea of the chapter, which exists in some parts of the chapter. The CNN is a feedforward network model structure based on artificial neural network. It has the advantages of local connection and weight sharing, which greatly reduces the number of parameters needed to learn in the network. Through multilayer nonlinear transformations, a CNN can learn the implicit features in a large-scale text [30]. So the AGFE based on CNN is designed to extract the core features *h*_*a*_ of a chapter-level text.

As shown in Figure 2(c), the AGFE consists of three one-dimensional convolutional layers, a maximum pooling layer, and a fully connected layer. Firstly, each one-dimensional convolution operation can obtain the features of the corresponding text fragment, and then after maximum pooling and fully connected operation with dropout, the final output of the AGFE *h*_*a*_ is obtained.

### 3.3. Seed of Entity Distribution Features

Different types of TCM texts cover different types of entities, and the distribution of entity label sequences reflects different patterns, which can enhance the effectiveness of entity label generation. The model proposed in this paper can obtain entity tagging distribution features based on the annotated corpus, which we call the seed of entity distribution features.

We refer to the classification criteria of the 12 first-level categories given in the *General catalogue of Ancient Chinese Medicine books* and select three basic categories of Chinese medical texts, with the additional category of comprehensive Chinese medical texts as the research object, then analyze the entities contained in above 4 categories of texts to summarize 19 typical entity types in total as shown in Table 1.

**Figure 3:**
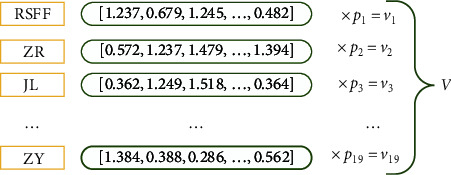
A schematic representation of entity distribution features.

As shown in Figure 3, the following are the construction steps of the seed for a certain type of TCM text.

  Step 1: Using word embeddings of 19 typical entities to generate 19 initial vectors *v*_*i*_, (*i*=1 ~ 19) independent of text type.
  Step2: Let *f*_*i*_(*i*=1 ~ 19) be the number of times entity type *i* occurs in the annotated corpus, and use *f*_*i*_ to calculate the probability distribution of each type of entity *p*_*i*_, (*i*=1 ~ 19). The calculated probabilities are then multiplied with the corresponding initial entity vectors *v*_*i*_(*i*=1 ~ 19) and spliced into a vector as the representation that reflects the distribution features of the entities in this corpus, which is used as the input to the Label Seed Feature Encoder (LSFE).

### 3.4. The Generator of the MT-CGAN

**Figure 4:**
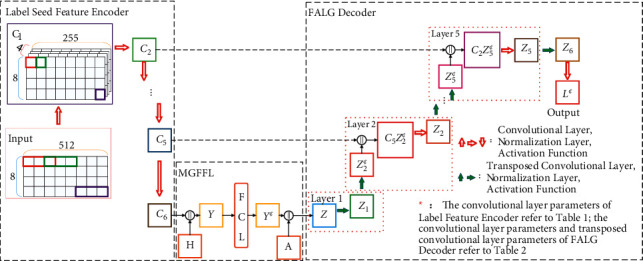
The structure diagram of the C-U-NET. FCL represents a fully connected layer.

In Figure 4, the *H* represents text features and the *A* represents sentence and paragraph-grained text attention features, both generated by the MTFE. Red arrows indicate convolutional layers (including Convolution, Normalization, and activation). Green arrows indicate transposed convolutional layers (including Transpose Convolution, Normalize, and Activate).

#### 3.4.1. The Generator Overview

The generator of the MT-CGAN consists of the MTFE and C-U-NET, and it is used to generate the sequence of entity labels corresponding to TCM texts. The C-U-NET is composed of the LSFE, the Multi-Granular Feature Fusion Layer (MGFFL), and the Feature Augmented Label Generation Decoder (FALG Decoder). The C-U-NET model is the main framework of the generator network. Overall, the encoding and decoding processes of the generator form a hierarchically symmetrical U-shaped structure as shown in Figure 4.

The encoder of the generator consists of the MTFE and LSFE of the C-U-NET. The role of the LSFE is to encode the features of seeds that reflect the distribution patterns of different categories of TCM entities and to output them hierarchically to the decoder, as shown in Figure 4. The decoder of the C-U-NET acts as the decoder of the generator. There is no data from other spaces introduced between the encoder and decoder of the traditional U-NET [31]. By adding a MGFFL in the middle of its encoder and decoder, we have improved the U-NET to form the C-U-NET.

#### 3.4.2. The C-U-NET

The encoding process of the encoder in the C-U-NET adopts a downsampling network structure. Each encoding layer passes the features extracted from that layer to the corresponding decoding layer, as shown in Figure 4. The decoding uses an upsampling network structure. The decoding process is enhanced by using feature information passed from the corresponding layer of the LSFE.

*(1) Label Seed Feature Encoder*. The LSFE is composed of 6 layers of CNN. There are a convolutional layer and a layer normalization and a ReLU activation function in each of the first five layers. The sixth layer consists of a convolutional layer and a layer normalization and a tanh activation function. The final layer uses tanh as the activation function to improve training efficiency and to preserve more fully the feature information of the labels.

*(2) FALG Decoder*. The role of the FALG Decoder is to decode feature sequences by convolution and transposed convolution, and it also contains 6 layers. The first layer is composed of a transposed convolutional layer, a layer normalization, and a ReLU activation function. Layers 2 to 5 consist of a transposed convolutional layer, a convolutional layer, two layer normalizations, and two ReLU activation functions. While the 6th layer has one normalization layer only and one of the activation functions has been replaced with tanh.

### 3.5. The Adversarial Optimization of the Model

#### 3.5.1. The Discriminator

The discriminator is composed of 7 convolutional layers. A layer normalization and a LeakyReLU activation function are added after each layer of convolution, and the Sigmoid activation function is used in the last layer. The LeakyReLU activation function maximizes the retention of features contained in the data. The Sigmoid, as a saturated activation function, is placed at the last layer of the network to compress the range of real values of the model and improve the accuracy of the discriminator. To meet the requirements of CGAN, the conditional and primary inputs of the discriminator are the real label sequence feature *L* and the generated label sequence feature *L*′, respectively.

#### 3.5.2. Confrontation Loss Function

To improve the generator accuracy of named entity label sequences, the generator and discriminator with different constraints as input are constructed. The CGAN converts the unsupervised generator model into a supervised or semisupervised generator model and converts the GAN loss probability representation into a conditional probability formula using conditions as restrictions. The discriminator optimizes the generator in terms of the truthfulness of the generated labels. The loss function is as in the following:

(10) $$\begin{array}{c} \underset{G}{\min}\underset{D}{\max}V\left(D,G\right)=E_{L∼P_{\text{data}}\left(L\right)}\left[\log\;D\left(L^{\prime }|L\right)\right]+E_{Y^{\prime }∼P_{Y^{\prime }}\left(Y^{\prime }\right)}\left[\log\left(1-D\left(G\left(A|Y^{\prime }\right)\right)\right)\right], \end{array}$$

where *D* is the discriminator, *G* is the generator, *𝔼* is the expected value of the sample, *𝔼*_*L*∼*P*_data_(*L*)_ is the data sample from the real data, *𝔼*_*Y*′∼*P*_*Y*′_(*Y*′)_ is the sample from the training data. The loss function of the MT-CGAN is as follows:

(11) $$\begin{array}{c} ℒ\left(\tilde{T}\right)={ℒ}_{\text{Cross Entropy}}\left(\underset{G}{\min}\underset{D}{\max}V\left(D,G\right)\right). \end{array}$$

where $\tilde{T}$ is the sequence of entity labels generated by the generator and ℒ_CrossEntropy_ is the cross-entropy loss function.

#### 3.5.3. Adversarial Training Process

As shown in Algorithm 1, the training of the MT-CGAN is divided into two steps. First, the generator is fixed and the discriminator is trained. Then, the discriminator is fixed and the generator is trained. In the training process, the two steps interact alternatively until reaching a stable state.

## 4. Experimental Analysis

### 4.1. Model Parameters and Datasets

  Experimental parameters. The MT-CGAN is implemented using Pytorch 1.3.1 library. The Adam optimizer is utilized during the training procedure to solve the problem of gradient sparsity and gradient oscillation in the field of natural language. The learning rate of the generator and discriminator is set to 1e − 4, the learning rate decay is 0.95, the dropout is 0.3, and the training epoch is set to 20. All models are trained and tested on a single NVIDIA RTX 3090.
  Datasets. We use four books Shennong's Classic of Material Medical, Medical Cases of Famous Doctors in Different Periods of China, Miraculous Pivot, and Syndrome in TCM, as the original texts for NER annotation and annotate four types of the corpus as the experimental dataset. The scale of the annotated corpus for each book is shown in Table 2.

The *Shennong's Classic of Material Medical* is one of the four classics of Chinese medicine, was written in the Han Dynasty, and is the earliest known book on herbals, containing 365 species of herb-medicine. The *Medical Cases of Famous Doctors in Different Periods of China* is a collection of Chinese medical cases recorded by famous doctors from all periods of Chinese history. There are more than 18,000 medical cases in total, and each case introduces the patient's illness involving the symptoms, pulse, tongue like, and the process of seeking medical treatment. The *Miraculous Pivot*, as an important part of the *Inner Canon of Huangdi*, is the earliest extant medical canon in China. The *Miraculous Pivot* is regarded as the most important classic that has summarized the theory of Jingluo (Channels and Collaterals) and the techniques of acupuncture before the Han Dynasty and contains 9 volumes and 81 chapters. The *Syndrome in TCM* is a comprehensive TCM book formed by collecting and sorting out ancient and modern documents related to medical writings, medical notes and medical cases.

### 4.2. Experiments and Results

For the TCM NER task, we chose several typical methods with which to compare the proposed approach. We selected two advanced methods: the BiLSTM-CRF architecture [4], BERT-BiLSTM-CRF architecture [13], and a variant of the BERT model architecture that can fuse multigranularity information of text (Roberta-c) [33]. These methods are described as follows.

BiLSTM-CRF [4] consists of the LSTM and CRF and is one of the most popular model architectures for handling NER tasks before pretraining models were proposed.

BERT-BiLSTM-CRF [13] consists of BERT, BiLSTM and CRF. As a representative of pretrained models, BERT has proven to be one of the most advanced models for representation learning in NLP [32]. The combination of BERT and BiLSTM-CRF models is one of the current popular architectures for NER task models.

Roberta-c [33] is a variant of the BERT model and outperforms the BERT model in Chinese NLP tasks. C denotes a word-character set in a self-attention module and it can integrate character and word information.

This section shows the comparative experiments and results analysis of the MGFF-GGAN with other three different types of baseline models on the datasets. The experimental results use Precision (P), Recall (R), and F1 score (F1) as evaluation metrics for the model evaluation. The performance on the test set of the different methods is presented in Tables 3 and 4.

According to the data presented in Tables 3 and 4, the overall performance of the proposed model is superior to that of the baseline models, except for on the *Medical Cases of Famous Doctors in Different Periods of China* dataset. This shows that our model shows overall advantages in various types of TCM texts, which is also the strongest advantage of our proposed model. Although the performance of the MT-CGAN does not improve significantly on both the *Shennong's Classic of Material Medical* and the *Medical Cases of Famous Doctors in Different Periods of China* datasets, even the effect of the F1 value on the *Medical Cases of Famous Doctors in Different Periods of China* dataset decreased by 0.08%, but the precision rate of the MT-CGAN is the highest. And as shown in Table 3 and Table 4, the *P* values of the proposed model are 90.26%, 90.36%, 77.73%, and 78.45%, respectively, on the four data sets, all of which are higher than other comparison models. It benefits from the fact that the MT-CGAN can extract the contextual semantic relations of text at different granularities.

We also observed that the performance of MT-CGAN on both *Miraculous Pivot* and *Syndrome in TCM* datasets was significantly improved. Especially on the *Syndrome in TCM*, the values of F1 and P of our proposed method are improved by 2.35∼5.70% and 3.83∼61.2%, respectively. The results indicate that the proposed model has good performance under the condition of small-scale annotated corpus and independent of external resources.

## 5. Discussion

### 5.1. Ablation Study

In this section, we investigate the ablation in different aspects of the MT-CGAN to better understand their relative importance.

We used the annotated corpus of the *Syndrome in TCM* as dataset to evaluate combination strategies at different granularities. As shown in Table 5, the F1 value is the highest when the features of the four granularities are used as input together. The F1 value is significantly improved when adding character-granularity features. The main reason is that the MT-CGAN uses GRU to extract the relative position features among characters from the character-granularity, which is used to constrain the order of entity label generation and plays a decisive role in the model.

We conducted a comparative experiment on whether the seed for different types of TCM texts can improve the effectiveness of named entity recognition. The results of the experiment, which are shown in Table 6, show that our method can enhance the effectiveness of entity label generation. When random entity label features are used as input, the F1 value is 75.16. In contrast, the F1 value increased by 2.19 when we used the seed of the corresponding text type as input.

### 5.2. Comparative Experiments with Different Forms of Main and Conditional Input

We verified the validity of our approach by experimentally comparing the differences with CGAN on the annotated corpus dataset of the *Syndrome in TCM* from two aspects. (1) Whereas the main input to a CGAN generator is white noise, the main input to the MT-CGAN generator is the attention data of a text. (2) While the generator and discriminator of CGAN use the same data as conditional inputs, the MT-CGAN uses different data as conditional inputs, respectively.

As shown in Table 7, the F1 value of the model was improved by 5.73% using the attention data of the text compared to white noise as the main input to the generator. The experimental results validate that the use of textual attention data can guide the model to generate more accurate labels.

As shown in Table 8, compared to the same conditional input, the F1 value of the MT-CGAN is improved by 4.56% and the number of training rounds for the model to reach the steady state is reduced by about 5 epochs. The experimental results demonstrate the method that uses the fused data *Y* as the conditional input in the model generator can improve the accuracy of the NER and speed up the convergence of the model.

### 5.3. The Comparison of Model Performance on Different Scales of Annotated Corpus

**Figure 5:**
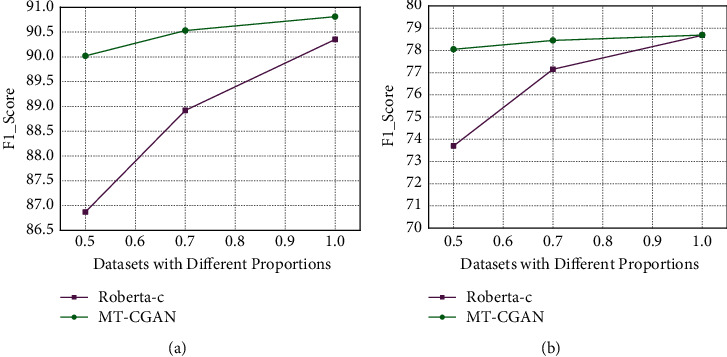
Comparison of experimental results on different corpus scale. (a) On *miraculous pivot* dataset (b) on *medical cases of famous doctors in different periods of China* dataset.

In this paper, we verified the performance of the MT-CGAN with small-scale samples by reducing the scale of the training dataset. We conducted comparison experiments with the Roberta-c model on different proportions of the original annotated datasets of the *Medical Cases of Famous Doctors in Different Periods of China* and *Miraculous Pivot,* respectively. As shown in Figure 5, we found that the MT-CGAN can maintain relatively stable F1 values, which indicates that the MT-CGAN has better recognition effects than other baseline models on the small-scale annotated corpus. The effectiveness of the MT-CGAN based on generative adversarial ideas for small-scale sample NER tasks was demonstrated.

## 6. Conclusions

In this paper, we propose the MT-CGAN to achieve TCM NER under the condition of a small-scale annotated corpus. To ensure that the model can learn multiple dimensions of TCM text information, MTFE is designed in MT-CGAN to extract text grammatical and semantic information from four granularity of words, sentences, paragraphs, and chapters. Moreover, to make MT-CGAN get accurate named entity labels when processing different types of TCM texts, the seed of entity distribution features is introduced as the input of the model. We verify MT-CGAN performance on different types of TCM texts corpora, and the experimental results show that the model can achieve accurate NER tasks. The main advantage of the MT-CGAN is to deal with texts that contain more entity types and has sparse entity distribution, and have a more random and less regular linguistic form. Although our approach outperforms previous methods, our model still fails in some situations. For example, the *R* and F1 scores of our experimental results are lower than those of certain models, so we will make efforts to improve the *R* and F1 values of the model in the future.

## Figures and Tables

**Algorithm 1:**
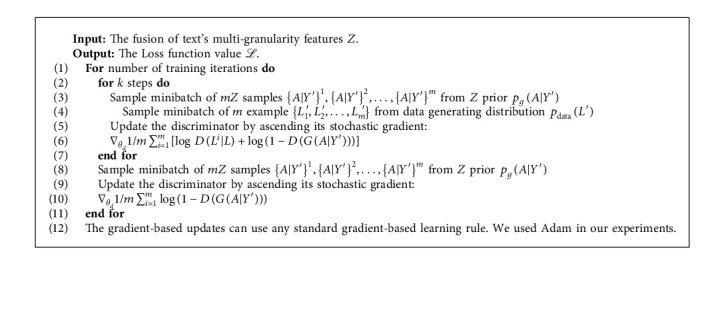
The training process. Minibatch stochastic gradient descent training of MT-CGAN. The number of steps to apply to the discriminator, *k*, is a hyperparameter. We used *k*=1. *Z* is the fusion of text's multigranularity features, seed of entity distribution features and attention data for different types of TCM texts.

**Table 1:** Typical types of entities contained in different types of TCM texts.

Type of literature	Typical entity types and corresponding labels
Canon of medicine	Cognitive method (RSFF), traditional Chinese physiology (ZYSL), traditional Chinese pathology (ZYBL), the principle of treatment (ZZ), method of treatment (ZF)
Medical cases	Chinese materia medica (ZY), symptoms (ZZ), pulse (MX), tongue (SX), formula (FJ), dosis (FJ)
Herbal	Drug property (YX), flavor of medicinals (YW), channel tropism (GJ), virtue (GX), dilantin (YM), symptoms (ZZ)
Comprehensive	Pathogeny (BY), disease (JB), syndrome (ZH), pulse (MX), tongue (SX), formula (FJ), Chinese materiamedica (ZY)

**Table 2:** The scale of annotated corpus of the experimental dataset.

Name	Type	Text size (10,000 words)	Size of annotated corpus (10,000 words)
*Shennong's classic of material medical*	Herbal	11.9	2.6
*Medical cases of famous doctors in different periods of China*	Medical cases	25	10
*Miraculous pivot*	Canon of medicine	7.9	7.9
*Syndrome in TCM*	General	564.4	28.6

**Table 3:** Experimental results under different models on the *Shennong's classic of material medical* and *medical cases of famous doctors in different periods of China* datasets.

Model	P (%)	R (%)	F1 (%)	P (%)	R (%)	F1 (%)
	*Shennong's classic of material medical*			*Medical cases of famous doctors in different periods of China*		
BiLSTM-CRF	86.32	87.67	86.99	87.88	89.43	88.65
BERT-BiLSTM-CRF	89.56	90.05	89.80	89.74	92.07	90.89
Roberta-c	89.78	90.15	89.96	90.12	90.59	90.35
MT-CGAN	90.26	89.78	90.02	90.36	91.28	90.81

**Table 4:** Experimental results under different models on the *Miraculous pivot* and *syndrome in TCM* datasets.

Model	P (%)	R (%)	F1 (%)	P (%)	R (%)	F1 (%)
	*Miraculous pivot*			*Syndrome in TCM*		
BiLSTM-CRF	68.76	66.95	67.85	72.38	70.95	71.65
BERT-BiLSTM-CRF	76.65	80.72	78.64	72.33	74.30	73.30
Roberta-c	76.98	80.47	78.68	74.62	75.39	75.00
MT-CGAN	77.73	79.69	78.69	78.45	76.28	77.35

**Table 5:** Experimental results of different combination strategies.

Combination strategies	F1 (%)
Sentence & paragraph	69.49
Sentence & paragraph &chapter	71.25
Character & sentence	74.86
Character & paragraph	73.17
Character & sentence & paragraph	76.48
Character & sentence & paragraph & chapter	77.35

**Table 6:** Comparison of using the seed of entity distribution features or not.

Label seed feature	F1 (%)
Random	75.16
Reflect entity distribution of different types of TCM text	77.35

**Table 7:** Experimental results of different main inputs.

Main input	F1 (%)
White noise	71.62
Attention data	77.35

**Table 8:** Experimental results of different condition inputs.

G	D	F1 (%)	Epoch
*L*	*L*	72.79	20–25
*Y*	*L*	77.35	15–20

*L*: the sequence of named entities labels for the corresponding text sequence. *Y*: the fusion of multigranularity text features and the seed of entity distribution features of the corresponding type of TCM text.

## Data Availability

Some data can be found within the article. Other data can be obtained from the corresponding author upon request.
